# Identification of special key genes for alcohol-related hepatocellular carcinoma through bioinformatic analysis

**DOI:** 10.7717/peerj.6375

**Published:** 2019-02-06

**Authors:** Xiuzhi Zhang, Chunyan Kang, Ningning Li, Xiaoli Liu, Jinzhong Zhang, Fenglan Gao, Liping Dai

**Affiliations:** 1 Henan Medical College, Zhengzhou, China; 2 Henan Province People's Hospital, Zhengzhou, China; 3 Institute of Medical and Pharmaceutical Sciences, Zhengzhou University, Zhengzhou, China

**Keywords:** Alcohol-related hepatocellular carcinoma, CD5L, CSMD1, Prognosis, Bioinformatic analysis

## Abstract

**Background:**

Alcohol-related hepatocellular carcinoma (HCC) was reported to be diagnosed at a later stage, but the mechanism was unknown. This study aimed to identify special key genes (SKGs) during alcohol-related HCC development and progression.

**Methods:**

The mRNA data of 369 HCC patients and the clinical information were downloaded from the Cancer Genome Atlas project (TCGA). The 310 patients with certain HCC-related risk factors were included for analysis and divided into seven groups according to the risk factors. Survival analyses were applied for the HCC patients of different groups. The patients with hepatitis B virus or hepatitis C virus infection only were combined into the HCC-V group for further analysis. The differentially expressed genes (DEGs) between the HCCs with alcohol consumption only (HCC-A) and HCC-V tumors were identified through limma package in R with cutoff criteria│log2 fold change (logFC)|>1.0 and *p* < 0.05. The DEGs between eight alcohol-related HCCs and their paired normal livers of GSE59259 from the Gene Expression Omnibus (GEO) were identified through GEO2R (a built-in tool in GEO database) with cutoff criteria |logFC|> 2.0 and *adj.p* < 0.05. The intersection of the two sets of DEGs was considered SKGs which were then investigated for their specificity through comparisons between HCC-A and other four HCC groups. The SKGs were analyzed for their correlations with HCC-A stage and grade and their prognostic power for HCC-A patients. The expressional differences of the SKGs in the HCCs in whole were also investigated through Gene Expression Profiling Interactive Analysis (GEPIA). The SKGs in HCC were validated through Oncomine database analysis.

**Results:**

Pathological stage is an independent prognostic factor for HCC patients. HCC-A patients were diagnosed later than HCC patients with other risk factors. Ten SKGs were identified and nine of them were confirmed for their differences in paired samples of HCC-A patients. Three (SLC22A10, CD5L, and UROC1) and four (SLC22A10, UROC1, CSAG3, and CSMD1) confirmed genes were correlated with HCC-A stage and grade, respectively. SPP2 had a lower trend in HCC-A tumors and was negatively correlated with HCC-A stage and grade. The SKGs each was differentially expressed between HCC-A and at least one of other HCC groups. CD5L was identified to be favorable prognostic factor for overall survival while CSMD1 unfavorable prognostic factor for disease-free survival for HCC-A patients and HCC patients in whole. Through Oncomine database, the dysregulations of the SKGs in HCC and their clinical significance were confirmed.

**Conclusion:**

The poor prognosis of HCC-A patients might be due to their later diagnosis. The SKGs, especially the four stage-correlated genes (CD5L, SLC22A10, UROC1, and SPP2) might play important roles in HCC development, especially alcohol-related HCC development and progression. CD5L might be useful for overall survival and CSMD1 for disease-free survival predication in HCC, especially alcohol-related HCC.

## Introduction

As one of the most common malignancies worldwide, the prognosis of hepatocellular carcinoma (HCC) is very poor, which lead to its role of the second leading cause of cancer death (Ferlay et al., 2015). The precursors of HCC are mainly liver cirrhosis, which is caused by many different risk factors including hepatitis B virus (HBV) infection, hepatitis C virus (HCV) infection, alcohol abuse, non-alcoholic fatty liver disease, and some other liver diseases. The HBV and HCV infections and alcohol abuse are the most important causes for HCC worldwide. Considering the efficacy improvements of hepatitis virus treatments and the alcohol consumption increase in many regions (Organization, 2014), alcohol is likely to become a leading role of HCC in the future. Recently, alcohol-related HCC was reported to be diagnosed in a later stage than non-alcohol related HCC (Bucci et al., 2016; Costentin et al., 2018; Schütte et al., 2012). As early detection is very important for tumor treatment, the prognosis would be poor when diagnosed later. Since there are no effective markers for its detection and prognosis prediction, identification of new molecular markers for alcohol-related HCC is very crucial.

With the development of microarray technology, it is easier to identify general genetic alterations and their functions in the progression of many tumors. In fact, some cancer-testis genes/gene families were reported (http://www.cancerimmunity.org/CTdatabase/). Among them, the melanoma antigen gene family (MAGE) and the chondrosarcoma associated gene family (CSAG) were shown to be frequently activated in many tumors (Yao et al., 2014). In HCC, high frequency MAGE genes expressions were reported (Chen et al., 1999; Kobayashi et al., 2000) and MAGEA1 and MAGEA3 were shown to be tumor-specific markers to detect blood dissemination of HCC cells (Mou et al., 2002). CSAG genes were also found to be up-regulated in HCC and CSAG1 over-expression was found to associated with the proliferation of HCC cells (Zong et al., 2009).

In recent years, GPC-3 and ACTL6A were shown to be new markers for HCC and their over-expressions in HCC were found to be associated with poor survival of the patients (Haruyama & Kataoka, 2016; Xiao et al., 2016). In contrast, some other genes were shown to be down-regulated in HCC or have anti-tumor effects during HCC development. Several solute carrier family 22 members including SLC22A1, SLC22A3, and SLC22A7 were shown to be under-expressed in HCC and their down-regulation was associated with the poor prognosis of HCC patients (Heise et al., 2012; Schaeffeler et al., 2011; Yasui et al., 2014). CD5 molecule-like (CD5L), also named inhibitor of microphage (AIM), was reported to have potent preventive effect on HCC (Maehara et al., 2014; Ozawa et al., 2016). It is noteworthy that although all these studies were about HCC, none of them were about alcohol-related HCC. Only one study (Udali et al., 2015) was about the gene expression profiles of alcohol-related HCC while no gene markers for its prognosis and progression were reported.

In the present study, two existing database, the Cancer Genome Atlas (TCGA, http://cancergenome.nih.gov) project (Wang, Jensen & Zenklusen, 2016) and the Gene Expression Omnibus (GEO, https://www.ncbi.nlm.nih.gov/geo/) database (Barrett et al., 2010), were used to find key genes during the process of alcohol-related HCC development. For the TCGA project, as one of the most useful cancer genomics programs, its relatively complete clinical information makes it easier for researchers to download and analyze the genomic data to find key genes of clinical significance for specific tumors. For the GEO database, as a public functional genomics data repository that includes array- and sequence-based data, it allows users to query and download experiments or gene expression profiles freely. Here, we identified differentially expressed genes (DEGs-1) between HCCs with alcohol consumption (only) and HCCs with one hepatitis virus (HBV or HCV) infection (only) through TCGA data analysis. The DEGs-1 and the differentially expressed genes (DEGs-2) between alcohol-related HCCs and their paired adjacent normal liver tissues by analyzing the mRNA data of GSE59259 from the GEO database were intersected to identify the important genes during alcohol-related development with higher specificity. The clinical significance of these genes was then further analyzed. These key genes, especially the ones with significant clinical significance, would provide some clues for the mechanism of alcohol-related HCC development and might be useful markers for diagnosis and prognosis predication as well as therapeutic targets.

## Materials and methods

### Data processing and analysis of HCC patients from TCGA database

TCGA liver HCC mRNA data of 369 patients and their clinical information were downloaded for analysis. Their mRNA expression levels were TMM (the trimmed mean of M-values normalization method) (Robinson & Oshlack, 2010) normalized. The HCC-related risk factors including alcohol consumption, HBV infection, HCV infection, and other liver disease were extracted for each patient. According to the risk factors with them, the HCC patients were divided into different groups. As shown in Table 1, 91 cases had no history of risk factors (HCC-N group), 68 had the risk factor of alcohol consumption only (HCC-A group), 74 had the risk factor HBV infection only (HCC-B group), 32 had the risk factor of HCV infection only (HCC-C group), 20 had the risk factors of alcohol consumption and HBV infection (HCC-AB group), 14 had risk factors of alcohol consumption and HCV infection (HCC-AC group), 11 patients had the risk factor of non-alcoholic fatty liver disease (HCC-NAF group). For the other 59 HCC patients, 18 cases had risk factors of other liver diseases or different risk factor combinations and there were 41 patients for which risk factors were not available.

**Table 1:** Clinicopathological characteristics of HCC patients^#^ from TCGA database.

Variable	HCC-N (*n* = 91)	HCC-A (*n* = 68)	HCC-B (*n* = 74)	HCC-C (*n* = 32)	HCC-AB (*n* = 20)	HCC-AC (*n* = 14)	HCC-NAF (*n* = 11)	Total (*n* = 310)
Risk factors	None	Alcohol	HBV	HCV	HBV alcohol	HCV alcohol	Non-alcoholic fatty liver disease	
Age at diagnosis (year)								*p* = 0.001
<60	39(42.6%)	23(33.8%)	50(67.6%)	13(40.6%)	13(65.0%)	6(42.9%)	3(27.3%)	
≥60	52(57.4%)	45(66.2%)	24(32.4%)	19(59.4%)	7(35.0%)	8(57.1%)	8(72.7%)	
Gender								*p* = 1.305E-13
Male	32(35.2%)	57(83.8%)	57(77.3%)	25(78.1%)	20(100%)	13(92.9%)	5(45.5%)	
Female	59(64.8%)	11(16.2%)	17(22.7%)	7(21.9%)	0(0%)	1(7.1%)	6(54.5%)	
Pathologic stage								*p* = 0.010
Stage I	30(33.0%)	20(22.0%)	46(62.3%)	16(50.0%)	15(75.0%)	9(64.3%)	5(45.4%)	
Stage II	20(22.3%)	13(19.1%)	16(21.6%)	7(21.9%)	3(15%)	3(21.4%)	3(27.3%)	
Stage III	33(36.2%)	25(36.8%)	7(9.4%)	5(15.6%)	2(10%)	1(7.1%)	0(0%)	
Stage IV	1(1.1%)	1(1.5%)	2(2.7%)	0(0%)	0(0%)	0(0%)	0(0%)	
NA	7(7.7%)	7(10.3%)	3(4.0%)	4(12.5%)	0(10%)	1(7.1%)	3(27.3)	
Pathologic grade								*p* = 0.002
G1	13(14.3%)	10(14.7%)	6(8.1%)	7(21.9%)	5(25.0%)	4(28.6%)	2(18.2%)	
G2	46(50.5%)	38(55.9%)	27(36.5%)	13(40.6%)	13(65.0%)	7(50%)	8(72.7%)	
G3	27(29.7%)	19(27.9%)	33(44.6%)	9(28.1%)	2(10%)	3(21.4%)	1(9.1%)	
G4	1(1.1%)	0(0%)	7(9.4%)	0(0%)	0(0%)	0(0%)	0(0%)	
NA	4(4.4%)	1(1.5%)	1(1.4%)	3(9.4%)	0(0%)	0(0%)	0(0%)	
Family history of cancer								*p* = 1.598E-4
None	43(47.3%)	45(66.2%)	53(71.6%)	14(43.7%)	18(96.0%)	7(50.0%)	5(45.5%)	
Yes	42(46.1%)	12(17.6%)	14(18.9%)	7(21.9%)	2(4.0%)	6(42.9%)	6(54.5%)	
NA	6(6.6%)	11(16.2%)	7(9.5%)	11(34.4%)	0(0%)	1(7.1%)	0(0%)	

Notes:

Chi-Square tests were used for the comparisons of age, gender, pathological stage, pathological grade, and family history of cancer in different groups. For age, Chi-square = 22.828, DF = 6, *n* of valid cases = 310; for gender, Chi-square = 72.415, DF = 6, *n* of valid cases = 310; for pathological stage, Chi-square = 34.800, DF = 18, *n* of valid cases = 275; for pathological grade, Chi-square = 39.451, DF = 18, *n* of valid cases = 301; for family history of cancer, Chi-square = 26.771, DF = 6, *n* of valid cases = 274.

HCC-N, HCC patients with no risk factor; HCC-A, HCC patients with alcohol consumption only; HCC-B, HCC patients with HBV infection only; HCC-C, HCC patients with HCV infection only; HCC-AB, HCC patients with alcohol consumption and HBV infection; HCC-AC, HCC patients with alcohol consumption and HCV infection; HCC-NAF, HCC patients with non-alcoholic fatty disease; NA, not available; DF, degrees of freedom.

# For the 371 HCC patients from TCGA database, only the patients with certain risk factor(s) (*n* = 310) were shown.

To ensure the reliability of the results, only the seven groups (HCC-N, HCC-A, HCC-B, HCC-C, HCC-AB, HCC-AC, and HCC-NAF) with certain risk factor(s) and with a sample size greater than 10 were included in the subsequent analysis. The clinicopathological characteristics of the 310 patients in the seven groups were shown in Table 1.

In this study, one-way ANOVA analysis, Mann–Whitney *U* tests, independent-samples *T* test, paired samples *T* test, correlation analysis and all the survival analysis were applied through SPSS18.0 (Chicago, IL, USA), *p* < 0.05 was considered to be statistically significant.

### Survival analysis of HCC patients

Kaplan–Meier (K–M) survival analysis was applied to investigate the effects of the risk factors (grouping) on the overall survival and disease-free survival of HCC patients.

With five variables including family history of cancer, age at diagnosis, gender, pathological grade, and pathological stage as the covariates, multivariable Cox regression analysis (Forward Stepwise, Likelihood Ratio) were applied to find the independent factor(s) for overall survival and disease-free survival of HCC patients.

### Pathological stages comparison among different groups of HCCs

As pathological stage was found to be independent prognostic factor for overall survival and disease-free survival of HCC patients, the pathological stages of patients in different groups were investigated through non-parametric tests (K independent samples, Mann–Whitney *U* test). The pathological stage difference between HCC-A and other HCC groups were also evaluated through a Mann–Whitney *U* test (two independent samples).

### Identification of DEGs-1 (DEGs between HCC-A group and other HCC groups)

Through the comparisons above, patients in the HCC-A group was found to be diagnosed at a later stage than the HCC-B group, HCC-C group, HCC-AB group, HCC-AC group, and HCC-NAF group. To find the gene expressional difference resulted from different risk factors which might be correlated with HCC pathological stage, the gene expression profiles were compared between the HCC-A group and other HCC groups. To avoid confusion caused by multiple factors, here only the HCC-A group, HCC-B group, and HCC-C group were selected for analysis. The HCC-B group and HCC-C group together were hereinafter referred to as HCC-virus (HCC-V) group. The DEGs (DEGs-1) between HCC-A tumors and HCC-V tumors were identified with limma (Ritchie et al., 2015) package in R software. The cutoff criterion was *p* < 0.05 and |log2 fold change (logFC)| > 1.0.

### Identification of DEGs-2 (DEGs between alcohol-related HCCs and paired normal liver tissues from GEO database)

The microarray data of GSE59259 (Udali et al., 2015) were downloaded from the GEO database and the mRNA expression data were processed with GEO2R, a built-in tool in GEO database. Statistically significant DEGs (DEGs-2) between alcohol-related HCCs and paired adjacent normal liver samples were identified with the cutoff criterion |logFC|> 2.0 and *adj.p* < 0.05.

### Key genes (with higher specificity for alcohol-related HCC) identification

To indentify the special key genes (SKGs) for alcohol-related HCCs comparing with HCC-V tumors, the DEGs-1 and DEGs-2 were intersected through Venn graphs (http://bioinformatics.psb.ugent.be/webtools/Venn/). The expressions of the SKGs in seven HCC-A tumors and their normal liver samples from the same patients were investigated (although there were 68 HCC-A patients, only seven of them were found to have paired normal samples in the TCGA dataset) and compared through paired samples *T* test for further validation.

To investigate the specificity of the genes in other HCCs, the SKGs expressions were compared between HCC-A group and HCC-N group, HCC-AB group, HCC-AC group, and HCC-NAF group, respectively, through independent-samples *T* test.

### The clinical significance of the SKGs in HCC-A patients

The correlations between SKGs and the pathological stage and grade of HCC-A tumors were analyzed through Spearman correlation analysis, *p* < 0.05 was considered to be statistically significant.

The multivariable Cox regression analysis were applied to find independent prognostic factor(s) for overall survival and disease-free survival of HCC-A patients with gender, age at diagnosis, family history of cancer, and all the SKGs as covariates.

### Evaluation of the SKGs in HCCs (without grouping) from TCGA database

Considering the effects of risk factors on the tumors, expression differences of the SKGs might be obscured in the condition of not grouping. Based on this, the expression profiles of the SKGs in all the HCCs from TCGA (HCCs overall) and their matched normal liver controls were analyzed through Gene Expression Profiling Interactive Analysis (GEPIA) (Tang et al., 2017). During the analysis, |log2FC|> 0.5 and *p* < 0.05 was considered to be statistically significant.

Considering the pathological stage difference between the HCC-A group and HCC-V group, the SKGs which were correlated with the HCC-A pathological stage were analyzed for their expressional differences among HCCs (overall) of different pathological stages through GEPIA to investigate their clinical significance in HCCs (overall). The prognostic value of the independent prognostic factors for HCC-A patients was also investigated in the HCCs (overall), with the median expression as the threshold and *p* < 0.05 was considered to be significant.

### Validation of the SKGs in HCC datasets through the Oncomine database

The expressions of the SKGs were also investigated through the Oncomine database (https://www.oncomine.org). For the differential analysis between HCCs and the normal liver controls, the thresholds were set as follows: analysis type: cancer vs. normal; cancer type: HCC; sample type: clinical specimen; data type: mRNA. For the gene expressional differences among different stages, the filters were set as follows: cancer type: HCC; data type: mRNA; pathology subtype: stage; sample type: clinical specimen. The clinical significance of the SKGs in survival of HCC patients were also analyzed with the following filters: cancer type: HCC; data type: mRNA; clinical outcome: survival status; sample type: clinical specimen.

One-way ANOVA analysis was used for the comparisons among HCCs of different stages. Two independents *T* test was used for the comparisons between HCCs of different survival status. For all the analysis, *p* < 0.05 was considered to be statistically significant.

## Results

### Pathological stage determines the overall and disease-free survival of HCC patients

**Figure 1:**
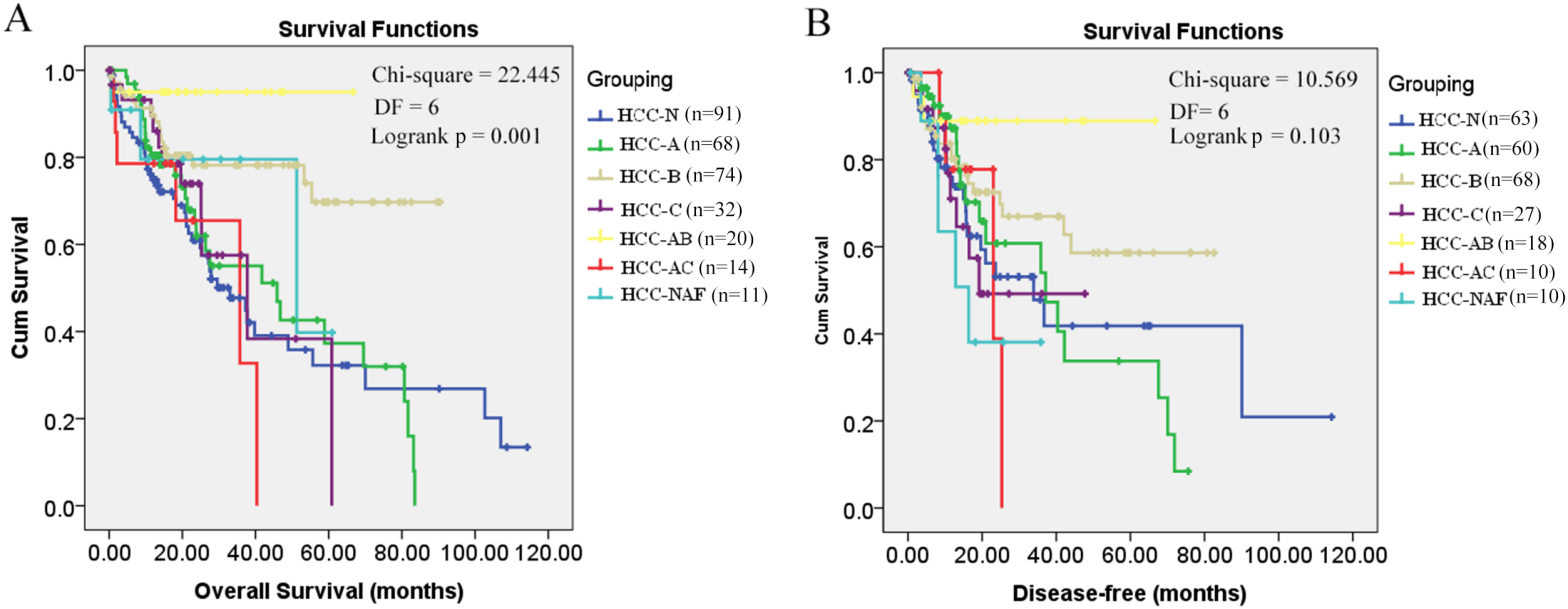
Kaplan–Meier survival analysis of HCC patients in different groups. (A) Overall survival analysis; (B) Disease-free survival analysis; *p* < 0.05 was considered to be statistically significant. HCC-N, HCC patients with no risk factor; HCC-A, HCC patients with alcohol consumption only; HCC-B, HCC patients with HBV infection only; HCC-C, HCC patients with HCV infection only; HCC-AB, HCC patients with alcohol consumption and HBV infection; HCC-AC, HCC patients with alcohol consumption and HCV infection; HCC-NAF, HCC patients with non-alcoholic fatty disease. DF, degrees of freedom.

**Figure 2:**
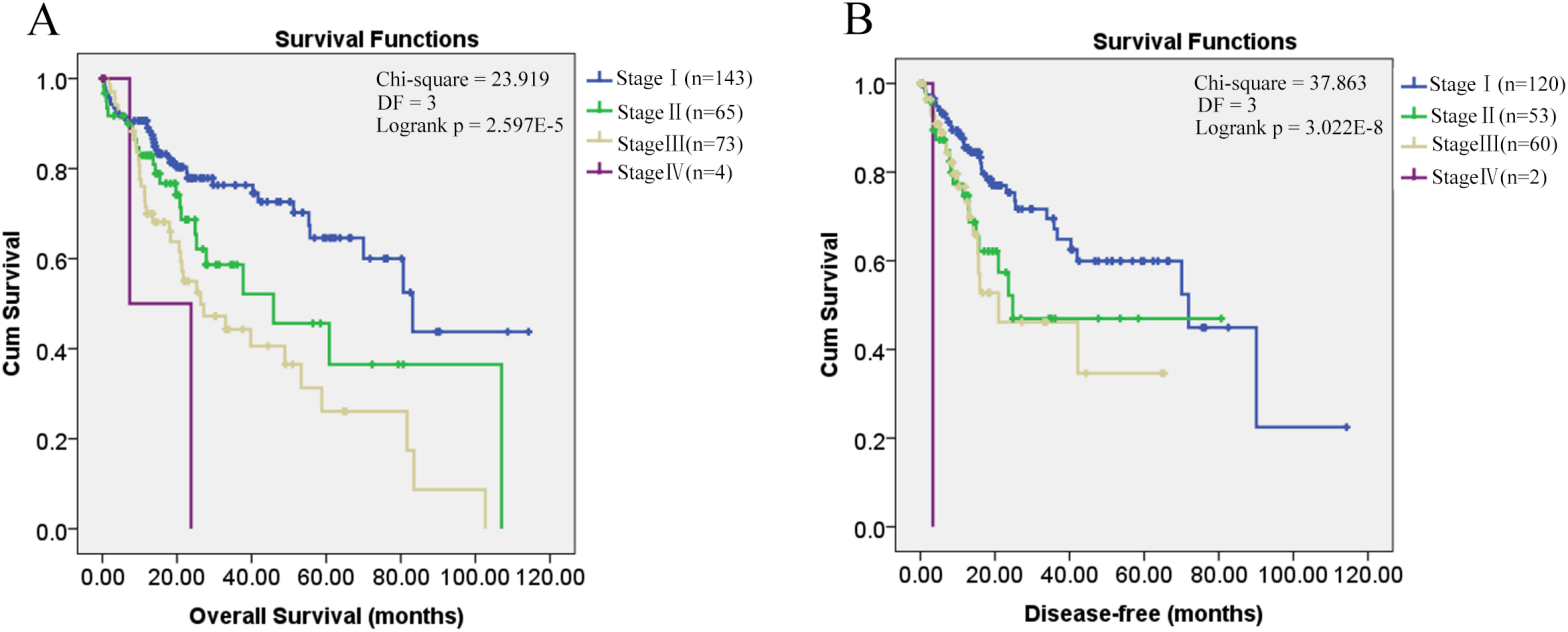
Kaplan–Meier survival analysis of HCC patients in different pathological stages. Kaplan–Meier survival analysis of HCC patients in different pathological stages. (A) Overall survival analysis; (B) Disease-free survival analysis; *p* < 0.05 was considered to be statistically significant. DF, degrees of freedom.

Through K-M survival analysis, the overall survival difference (*p* = 0.001, *p* < 0.05) among HCC patients of different groups was shown (Fig. 1A). With grouping as the strata variable, through multivariable Cox regression (stepwise) analysis, pathological stage was shown to be independent unfavorable prognostic factor with the hazard ratio (HR) 1.477 (95%CI, 1.147–1.902) for overall survival of HCC patients (Table 2). As no significant disease-free survival difference (*p* = 0.103, *p* > 0.05) was found among HCC patients of different groups (Fig. 1B), grouping was not used in the analysis of disease-free survival prognostic factor identification. Among the five variables mentioned above, only pathological stage was shown to be unfavorable independent prognostic factor for disease-free survival with the HR 1.861 (95%CI, 1.237–2.802) (Table 2). Through K-M survival analysis, the overall survival and disease-free survival difference among HCC patients of different pathological stages was visualized (Fig. 2).

**Table 2:** Cox regression analysis (stepwise) of the variables^#^ in HCC patients.

Survival analysis	Prognostic factor	B	SE	HR(95.0% CI)	*p*
Overall survival^§^	Pathological stage	0.390	0.129	1.477(1.147–1.902)	0.003
Disease-free survival	Pathological stage	0.331	0.153	1.392(1.031–1.880)	0.031

**Notes:**

B, regression coefficient; SE, standard error; HR, hazard ratio; CI, confidence interval. *p* < 0.05 was considered to be statistically significant.

# Five variables including family history of cancer, age at diagnosis, gender, pathological grade, and pathological stage as the covariates.

§ Grouping was used as strata variable in the analysis.

**Figure 3:**
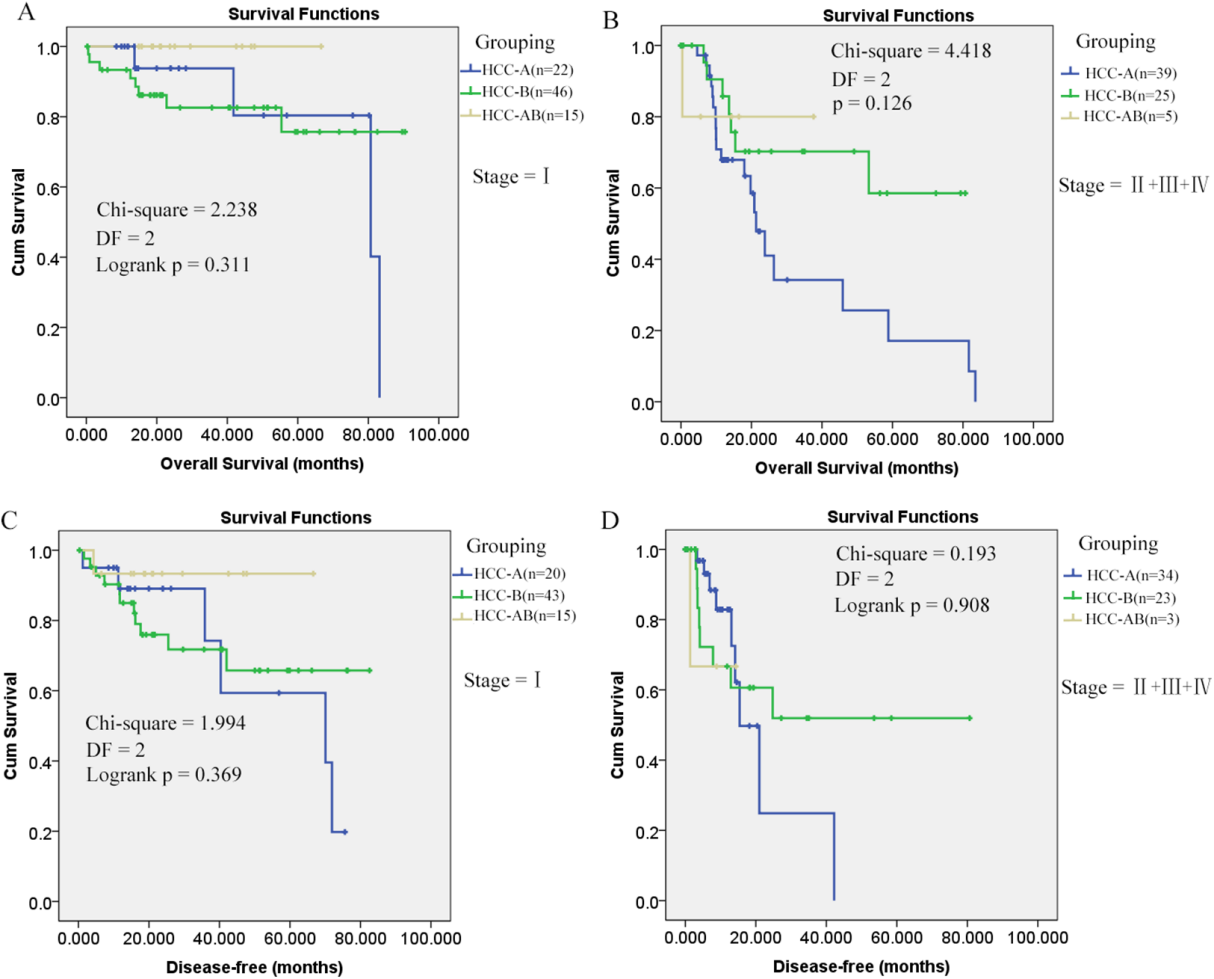
Overall and disease-free survival comparisons of HCC-AB, HCC-A, and HCC-B patients in the same stage. (A) and (B) Overall survival comparisons of HCC-A, HCC-B, and HCC-AB patients in stage I and stage II + III + IV , respectively. (C) and (D) Disease-free survival comparisons of HCC-A, HCC-B, and HCC-AB patients in stage I and stage II + III + IV , respectively. HCC-A, HCC patients with alcohol consumption only; HCC-B, HCC patients with HBV infection only; HCC-AB, HCC patients with alcohol consumption and HBV infection; DF, degrees of freedom. Kaplan–Meier survival analysis was used and *p* < 0.05 was considered to be statistically significant.

As shown in Fig. 1, it is interesting to see that HCC-AB group has the highest overall survival and disease-free survival. To further investigate the prognostic role of alcohol and HBV infection in HCC patients, the overall survival and disease-free survival were compared through K-M analysis among the three groups (HCC-A, HCC-B, and HCC-AB). As pathological stage was shown to be independent prognostic factor for the overall and disease-free survival of HCC patients in the above analysis, the overall and disease-free survival were compared in the patients of stage I and stage II+III+IV, respectively. But no significant difference was shown in the overall survival and disease-free survival among the three groups with pathological stage as the strata variable (*p* > 0.05) (Fig. 3), indicating that it was the pathological stage, not the alcohol consumption and HBV infection, which mainly accounted for the survival difference of the patients.

### HCC-A patients are diagnosed later than HCC patients with other risk factors

As shown in Table 3, through Mann–Whitney *U* tests for several independent samples, significant difference in pathological stage was shown among HCCs of the seven groups. Considering the independent role of pathological stage in the overall and disease-free survival, it is not surprising to see the survival difference among the patients of different groups. When Mann–Whitney *U* tests for two independent samples were applied, HCC-A patients were shown to be diagnosed at a later stage compared with HCC-B, HCC-C, HCC-AB, HCC-BC, and HCC-NAF patients. While no significant difference of pathological stage was found between HCC-N patients and HCC-A patients (Table 4).

**Table 3:** Comparison of pathological stages among different HCC groups.

Group	HCC-A	HCC-N	HCC-B	HCC-C	HCC-AB	HCC-AC	HCC-NAF	Chi-Square	DF	*p*
Mean rank	168.53	167.24	118.45	128.89	104.90	109.31	111.00	33.012	6	1.429E-5**

**Notes:**

Method: Mann–Whitney *U* tests for several independent samples. DF, degrees of freedom.

** *p* < 0.01; *p* < 0.05 was considered to be statistically significant.

**Table 4:** Comparison of pathological stage between HCC-A patients and patients in other HCC groups.

HCC-A vs.	HCC-N	HCC-B	HCC-C	HCC-AB	HCC-AC	HCC-NAF
Z	−0.145	−3.738	−2.279	−3.124	−2.501	−2.068
*p*	0.884	1.852E-4**	0.023*	0.002*	0.012*	0.039*

**Notes:**

Method: Mann–Whitney *U* tests for two independent samples.

*p < 0.05; **p < 0.01; *p* < 0.05 was considered to be statistically significant.

### Identification of DEGs-1 (DEGs between HCC-A tumor and other HCCs)

Considering the potential effects of mixed risk factors in the patients of HCC-AB group and HCC-AC group, only HCC-B patients and HCC-C patients were selected to compare with HCC-A patients for DEGs-1 identification. As no significant pathological stage difference (*p* = 0.473, *p* > 0.05) was found between HCC-B and HCC-C patients, the two groups were combined to be HCC-V group.

**Figure 4:**
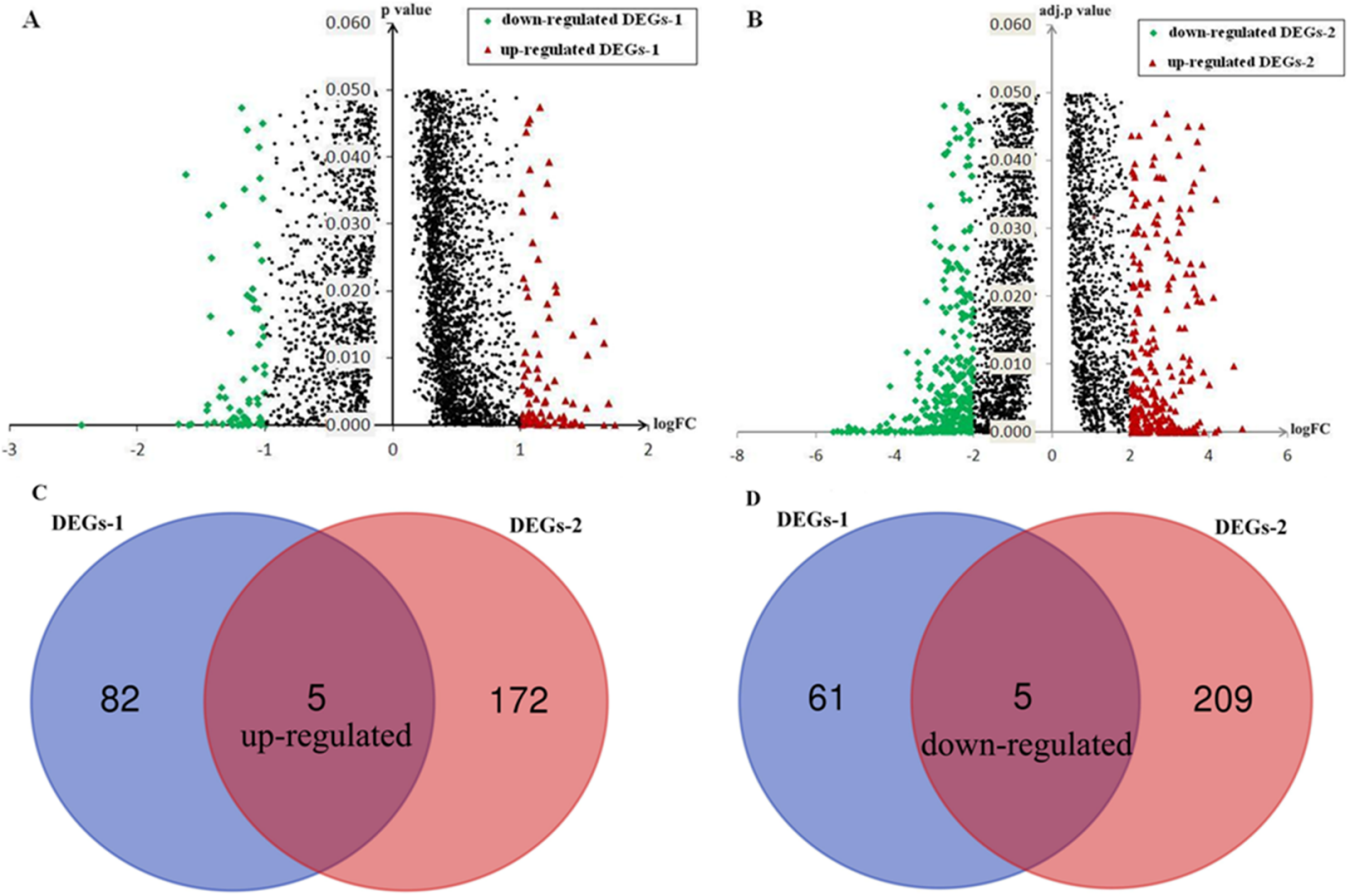
Identification of DEGs and SKGs. (A) DEGs-1(DEGs between HCC-A patients and HCC-V patients); (B) DEGs-2 (DEGs between alcohol-related HCCs and their paired normal liver controls); (C) Up-regulated SKGs; (D) Down-regulated SKGs. SKGs, special key genes for alcohol-related HCCs and HCC-A patients DEGs, differentially expressed genes; SKGs, special key genes; HCC-A, HCC patients with alcohol consumption only; HCC-V, HCC patients with HBV or HCV infection only.

After applying cutoff criteria, 153 genes (DEGs-1) including 87 up-regulated genes and 66 down-regulated genes in HCC-A HCCs were identified compared with HCC-V HCCs (Fig. 4A).

### Identification of DEGs-2 (DEGs between alcohol-related HCCs and paired normal liver tissues from the GEO database)

After applying cutoff criteria, 391genes (DEGs-2) including 177 up-regulated genes and 214 down-regulated genes in alcohol-related HCCs were identified compared with their paired normal liver tissues (Fig. 4B).

### SKGs identification

**Figure 5:**
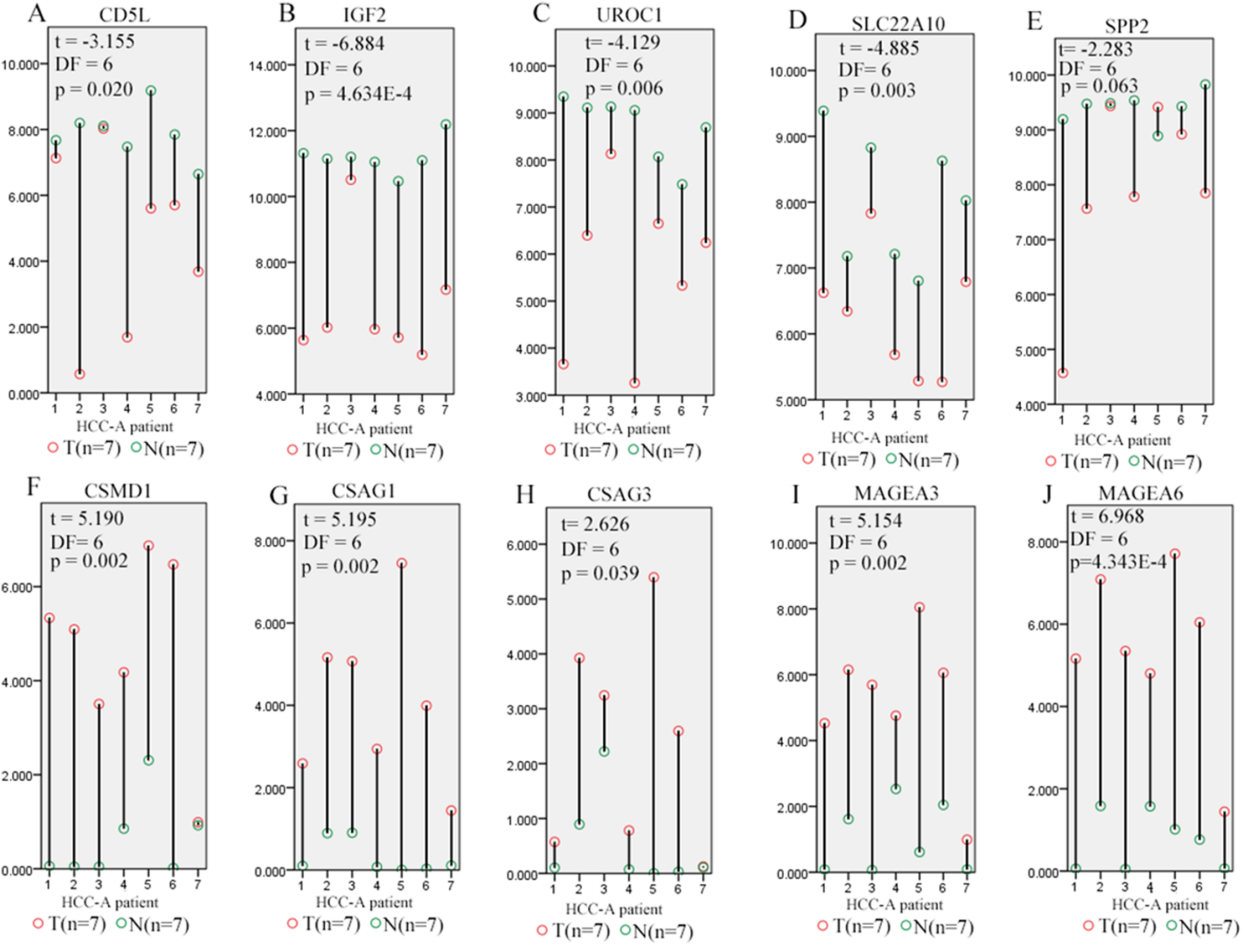
Differential expression profiles of SKGs in paired tumor and normal samples from the same HCC-A patients. (A–D) CD5L, IGF2, UROC1, and SLC22A10 were significant lower in HCC-A tumors than their paired normal livers from the same patients; (E) SPP2 had a lower trend (0.05 < *p* = 0.063 < 0.1) in HCC-A tumors than their paired normal livers; (F–J) CSMD1, CSAG1, CSAG3, MAGEA3, and MAGEA6 were significantly higher in HCC-A tumors than their paired normal livers from the same patients. *Y*-axis represented the relative expression of the genes (TMM normalized and log2(x+1) transformed) and the *x*-axis represented the seven HCC-A patients with paired tumor and liver samples. HCC-A, HCC patients with alcohol consumption only. Paired samples *T* test was used and *p* < 0.05 was considered to be statistically significant.

Through Venn graph analysis, five up-regulated genes and five down-regulated genes were identified which were considered to be SKGs for alcohol-related HCCs compared with HCC-V tumors (Figs. 4C and 4D). For the up-regulated SKGs, CUB and Sushi multiple domains 1 (CSMD1), MAGEA3, MAGEA6, CSAG1, and CSAG3 were included. For the down-regulated SKGs, CD5L, secreted phosphoprotein 2 (SPP2), urocanate hydratase 1 (UROC1), insulin-like growth factor 2 (IGF2), and solute carrier family 22 member 10 (SLC22A10) were included. Through paired samples *T* tests, nine of the SKGs including all the up-regulated genes and four of the down-regulated genes (CD5L, UROC1, IGF2, and SLC22A10) showed significant differences between the paired samples (Fig. 5). Although no significant difference of SPP2 was shown between the samples, its lower trend in the HCC-A tumors than their paired normal livers was obvious and five of the HCC-A tumors had higher SPP2 expression in the tumors than their paired normal controls (Fig. 5E).

For the five down-regulated genes, through Human Protein Atlas database (Uhlén et al., 2015) analysis, three (SPP2, UROC1, and SLC22A10) of them were shown to be liver enriched genes (https://www.proteinatlas.org/humanproteome/tissue/liver). Considering the correlations between tissue-specific genes and tissue differentiation (Guillemin, Guais & Francastel, 2007; Stein, Lian & Owen, 1990a; Stein, Lian & Owen, 1990b), we speculated that the down-regulation of the liver enriched genes might be associated with HCC differentiation or play some important roles in HCC progression.

In fact, as shown in Table 5, UROC1, and SLC22A10 were shown to be negatively correlated with the HCC-A pathological stage and pathological grade (*p* < 0.05). Down-regulated CD5L was also shown to be negatively with the HCC-A pathological stage while up-regulated CSAG3 and CSMD1 were shown to be positively correlated with the HCC-A pathological grade (*p* < 0.05). For SPP2, although the expressional difference between paired HCC-A tumor and liver samples were not confirmed to be so significant, its negative correlations with HCC-A stage and grade were obvious. Considering its lower trend in HCC-A tumors than their paired normal controls, SPP2 might play some important roles during HCC-A progression.

**Table 5:** Correlations analysis between SKGs and the pathological stage and grade of HCC-A tumors.

Variable	Correlation with pathological stage (*n* = 61)		Correlation with pathological grade (*n* = 67)	
	*R*	*p* value	*R*	*p* value
SLC22A10	−0.378	0.003**	−0.368	0.002**
CD5L	−0.333	0.009**	−0.160	0.196
UROC1	−0.320	0.012*	−0.346	0.004**
SPP2	−0.298	0.020*	−0.440	2.933E-7**
CSAG3	−0.162	0.211	0.289	0.018*
CSMD1	0.083	0.524	0.293	0.016*
MAGEA3	−0.062	0.635	0.138	0.266
IGF2	−0.032	0.806	−0.151	0.221
CSAG1	−0.025	0.850	0.214	0.082
MAGEA6	−0.006	0.963	0.204	0.099

**Notes:**

*R*, correlation coefficient; SKGs, special key genes.

*p < 0.05; **p < 0.01; *p* < 0.05 was considered to be statistically significant.

Comparing with other HCC groups, through independent samples *T* tests, IGF2 (*t* = −2.36, DF = 157, *p* = 0.02), SPP2 (equal variances not assumed, *t* = −3.030, DF = 45.723, *p* = 0.004), and SLC22A10 (*t* = −2.031, DF = 77, *p* = 0.046) were shown to be significantly lower in HCC-A than the HCC-N group, HCC-AC group, and the HCC-NAF group, respectively. While UROC1 was found to have a lower trend (0.05 < *p* < 0.1) in HCC-A group than HCC-AB (*t* = −1.669, DF =86, *p* = 0.099) group and HCC-NAF (*t* = −1.871, DF = 77, *p* = 0.065) group, CD5L was shown to have a lower trend (equal variances not assumed, *t* = −1.731, DF = 25.062, *p* = 0.096, 0.05 < *p* < 0.1) in HCC-A group than HCC-AC group. For the up-regulated genes, MAGEA3 (*t* = 1.817, DF = 157, *p* = 0.071), MAGEA6 (*t* = 1.818, DF= 157, *p* = 0.071), and CSAG3 (*t* = 1.849, DF = 157, *p* = 0.066) were shown to have a higher trend (0.05 < *p* < 0.1) in HCC-A group than HCC-N group while CSMD1 and CSAG1 showed no significant expressional difference between the HCC-A group and other HCC groups. As eight of the ten genes showed differential expressions or differential expression trends between HCC-A and at least one of the other four HCC groups, their higher specificity was indicated.

### Prognostic factors for overall and disease-free survival of HCC-A patients

To identify independent prognostic factor(s) for overall survival and disease-free survival of HCC-A patients, thirteen variables including the 10 genes in SKGs, gender, age at diagnosis, and family history of cancer were applied to multivariable stepwise Cox regression analysis for overall survival and disease-free survival. As shown in Table 6, through overall survival analysis, CD5L was shown to be independent favorable prognostic factor for HCC-A patients with the HR 0.814 (95%CI: 0.709–0.936). When it came to disease-free survival analysis, among the thirteen variables, only the gene CSMD1 was shown to be independent unfavorable prognostic factor for HCC-A patients with the HR 1.540 (95%CI: 1.204–1.970).

**Table 6:** Cox regression analysis^#^ for overall survival and disease-free survival in HCC-A patients.

Survival analysis	Strata variable	Prognostic factor	B	SE	HR(95.0% CI)	*p*
Overall survival	None	CD5L	−0.205	0.071	0.814(0.709–0.936)	0.004**
	Pathological stage	None				
Disease-free survival	None	CSMD1	0.432	0.126	1.540(1.204–1.970)	0.001**
	Pathological grade	CSMD1	0.315	0.123	1.370(1.076–1.744)	0.021*

**Notes:**

SKGs, special key genes; B, regression coefficient; SE, standard error; HR, hazard ratio; CI, confidence interval.

# Forward Stepwise (Likelihood Ratio) method was used for Cox regression analysis with 13 variables including the 10 genes in SKGs, gender, age at diagnosis, and family history of cancer as covariates.

*p < 0.05; **p < 0.01; *p* < 0.05 was considered to be statistically significant.

**Figure 6:**
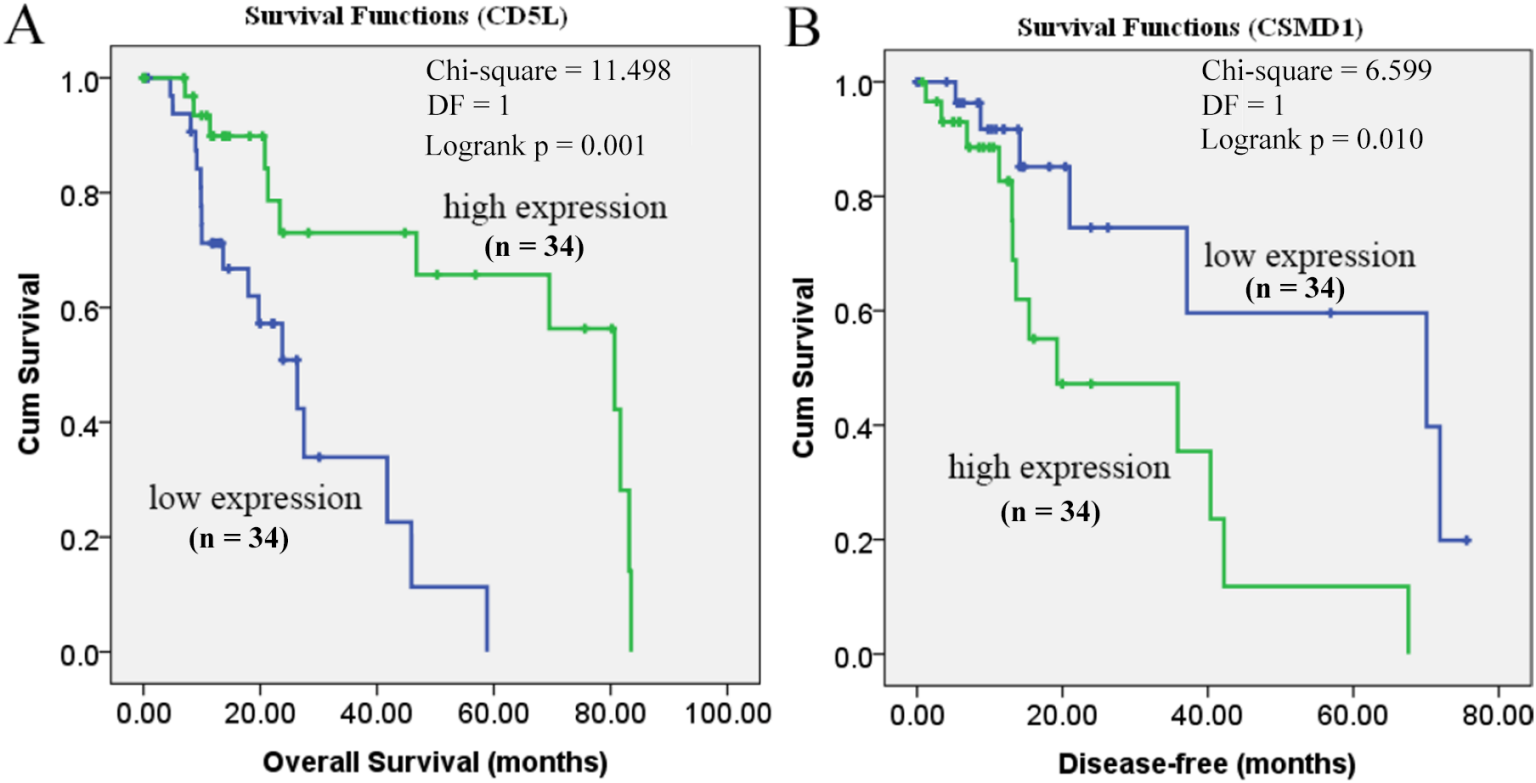
Prognostic roles of CD5L and CSMD1 in the survival of HCC-A patients. (A) Overall survival analysis of HCC-A patients with low and high CD5L expression; (B) Disease-free survival analysis of HCC-A patients with low and high CSMD1 expression. The median expression of CD5L and CSMD1 was set as the threshold, respectively, and there were 34 patients in each group (high expression group and low expression group). HCC-A, HCC patients with alcohol consumption only; DF, degrees of freedom. Kaplan–Meier analysis was used and *p* < 0.05 was considered to be statistically significant.

As shown in Fig. 6, the prognostic power of CD5L on overall survival and CSMD1 on disease-free survival in HCC-A patients were also visualized through the K-M analysis during which HCC-A patients were divided into high expression group and low expression group (with the median value of the gene expression as the threshold).

As CD5L was shown to be correlated with pathological stage and CSMD1 was shown to be correlated with pathological grade, multivariable stepwise Cox regression analysis with pathological stage as strata variable for overall survival analysis and pathological grade as strata variable for disease-free survival analysis were also applied. With the pathological stage as the strata variable, the prognostic power of CD5L disappeared (Table 6). It was indicated that the prognostic effect of CD5L on HCC-A patients might be resulted from its association with pathological stage. For CSMD1, although its positive correlation with pathological grade was shown above, its prognostic power still existed when pathological grade was set as strata variable (Table 6). In other words, the prognostic power of CSMD1 for disease-free survival in HCC-A patients might not depend on its association with pathological grade.

### Differential expression profiles of the SKGs in HCCs (without grouping, overall)

**Figure 7:**
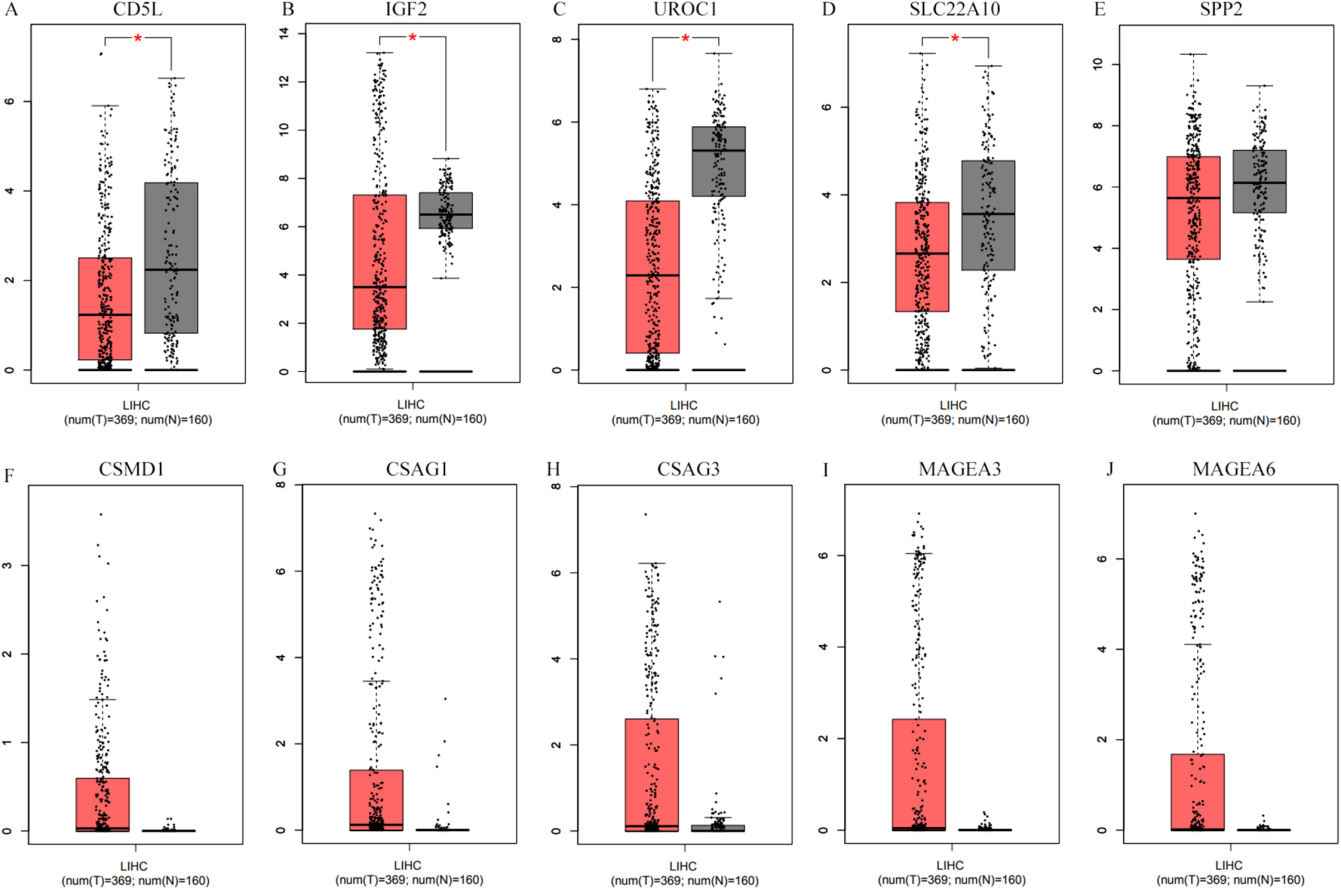
Differential expression analysis of the SKGs in HCCs (overall) and their normal liver controls through GEPIA. (A–E), down-regulated SKGs; (F–J), up-regulated SKGs. (A–D) indicated CD5L, IGF2, UROC1 and SLC22A10 were significantly down-regulated in HCCs patients, respectively. The expression data are first log_2_(TPM+1) transformed for differential analysis and the log2FC is defined as median(Tumor) − median(Normal). The threshold for log2FC was set at 0.5; *p* < 0.05 was considered to be statistically significant. LIHC, liver hepatocellular carcinoma. SKGs, special key genes.

As shown in Fig. 7, when matched TCGA normal and Genotype-Tissue Expression project normal liver data, after applying the cutoff criteria, only four down-regulated genes (CD5L, IGF2, UROC1, and SLC22A10) were shown to be down-regulated in HCCs. Considering differences of the other six genes between HCCs (overall) and the normal liver controls were not so significant, their differential profiles in paired samples of HCC-A patients might be obscured in this condition.

**Figure 8:**
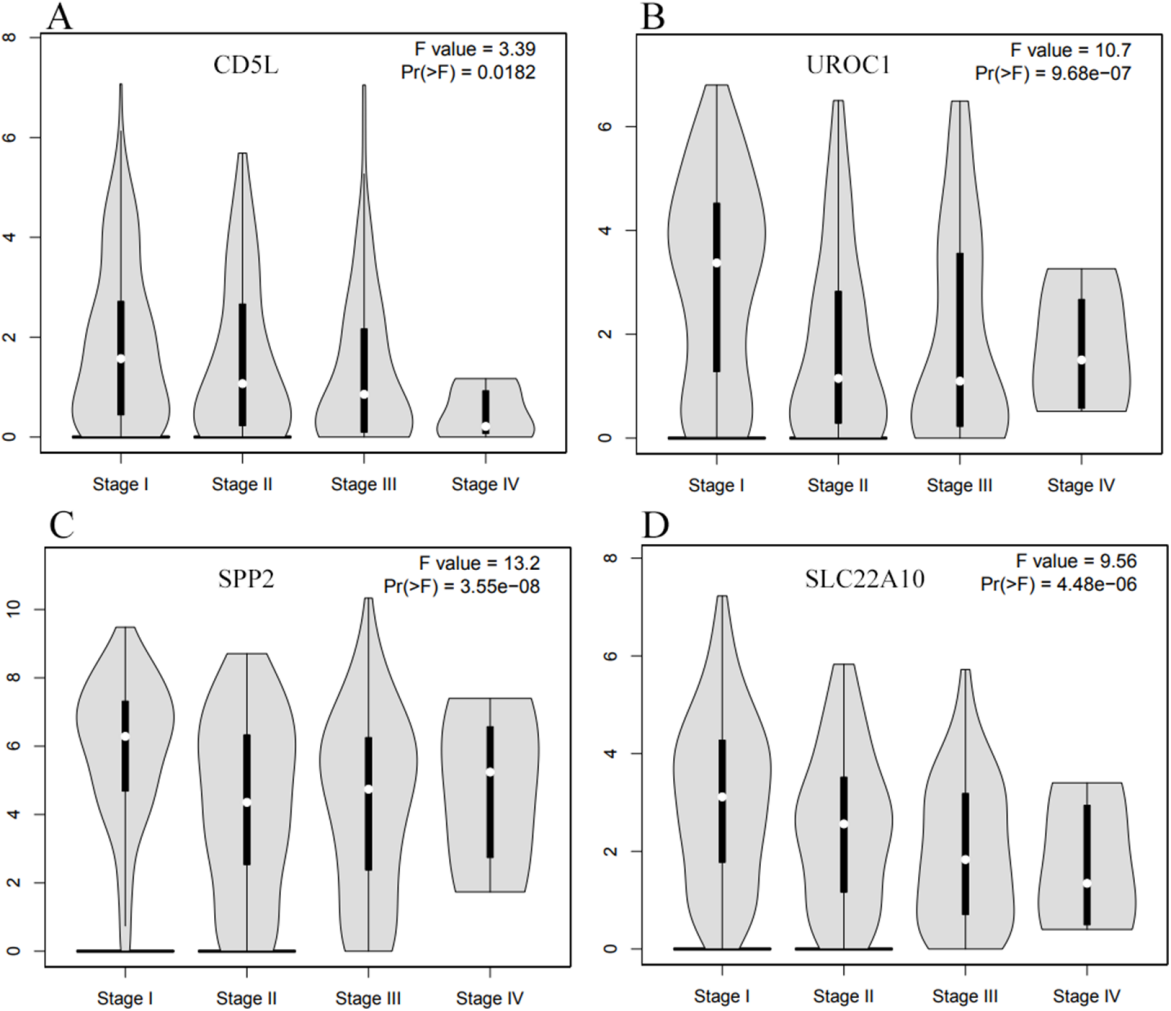
The differential expression profiles of the SKGs in HCCs (overall) of different stages through GEPIA. (A) CD5L is differentially expressed in HCCs of different stages; (B) UROC1 is differentially expressed in HCCs of different stages; (C) SPP2 is differentially expressed in HCCs of different stages; (D) SLC22A10 is differentially expressed in HCCs of different stages. One-way ANOVA was used *p* < 0.05 was considered to be statistically significant. SKGs, special key genes.

As CD5L, UROC1, SPP2, and SLC22A10 were shown to be correlated with HCC-A pathological stage, their expressions in HCCs (overall) of different stages were also analyzed through GEPIA. All the four genes showed their differential expression among HCCs (overall) of different pathological stages (Fig. 8). As the differential expression of SPP2 was not so significant between HCCs (overall) and the normal liver controls, their correlations with pathological stage might be neglected in the condition of not grouping, the importance of grouping the HCCs according to their risk factors was indicated.

**Figure 9:**
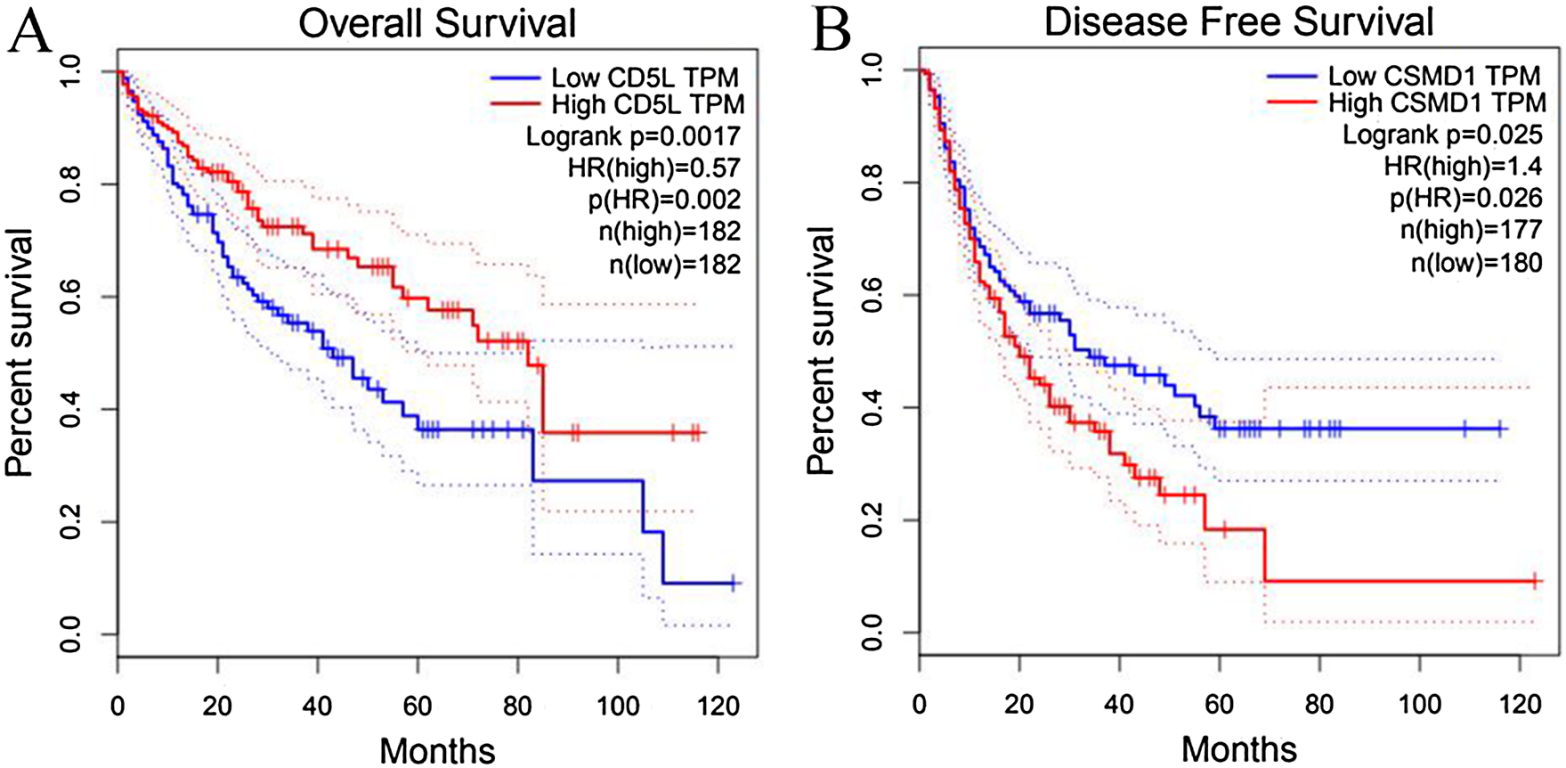
Survival analysis of CD5L and CSMD1 in all the HCC patients from TCGA database through GEPIA. (A) HCC patients high CD5L expression have a better overall survival than those with low expression; (B) HCC patients high CSMD1 expression have a worse disease-free survival than those with low expression. The median expression of CD5L and CSMD1 was set as the threshold for splitting the high-expression and low-expression cohorts. Log Rank (Mantel–Cox) was used and *p* < 0.05 was considered to be statistically significant.

Through survival analysis, CD5L was also shown to be favorable prognostic factor for overall survival and CSMD1 was shown to be unfavorable prognostic factor for disease-free survival in HCC patients (overall) (Fig. 9).

### Validation of SKGs through Oncomine database

For the differential analyses between HCCs and the normal liver controls, after applying the filters, five datasets including Chen liver (Chen et al., 2002), Mas liver (Mas et al., 2009), Roessler liver (Roessler et al., 2010), Roessler liver 2 (Roessler et al., 2010), and Wurmbach liver (Elisa et al., 2010) were selected and the SKGs were searched one by one. As shown in Table 7, although no data were found for three genes (SLC22A10, CSAG1, and CSAG3), the other seven genes were all found to be differentially expressed between HCCs and their normal liver controls in at least one of the datasets. CD5L was found to be down-regulated in four of the five datasets and IGF2 down-regulated in all the five datasets in HCCs comparing with the normal liver controls. The only one dataset with UROC1 expression data showed its down-regulation in HCCs while two datasets with CSMD1 expression data showed its up-regulation in HCCs. MAGEA3 and MAGEA6 were shown to be up-regulated in three of the four datasets with their expression data. No opposite results were found for the above six genes. However, one opposite result was found for SPP2 which was found to be up-regulated in HCCs in four of the five datasets while up-regulated in one dataset (Mas liver), indicating the expressional heterogeneity of SPP2 in HCC patients.

**Table 7:** Expressional differences of SKGs in HCC datasets from Oncomine database.

	Cancer vs. normal	Chen liver (*n* = 197)	Mas liver (*n* = 115)	Roessler liver (*n* = 43)	Roessler liver 2 (*n* = 445)	Wurmbach liver (*n* = 75)
CD5L	Fold change *p*	− 7.2759.22E-19**	1.0440.605	−5.1682.51E-18**	−5.3086.52E-95**	−6.7001.23E-9**
IGF2	Fold change *p*	−3.0801.32E-5**	−1.7352.25E-4**	−3.1630.015*	−4.1543.43E-16**	−10.1202.27E-6**
UROC1	Fold change *p*	NA	NA	NA	NA	-2.1920.004**
SLC22A10	Fold change *p*	NA	NA	NA	NA	NA
SPP2	Fold change *p*	−4.9825.74E-15**	2.4827.60E-5**	−9.5813.27E-7**	−5.7901.18E-42**	−3.0860.006**
CSMD1	F*old change* *p*	1.2560.011*	NA	NA	NA	1.3140.034*
CSAG1	Fold change *p*	NA	NA	NA	NA	NA
CSAG3	Fold change *P*	NA	NA	NA	NA	NA
MAGEA3	Fold change *p*	NA	1.1130.139	2.4500.011*	2.7582.53E-17**	2.8330.001**
MAGEA6	Fold change *P*	NA	1.1650.094	2.4450.009**	2.5112.16E-17**	3.2172.73E-4**

**Notes:**

NA, not available.

*p < 0.05; **p < 0.01; *p* < 0.05 was considered to be statistically significant.

**Figure 10:**
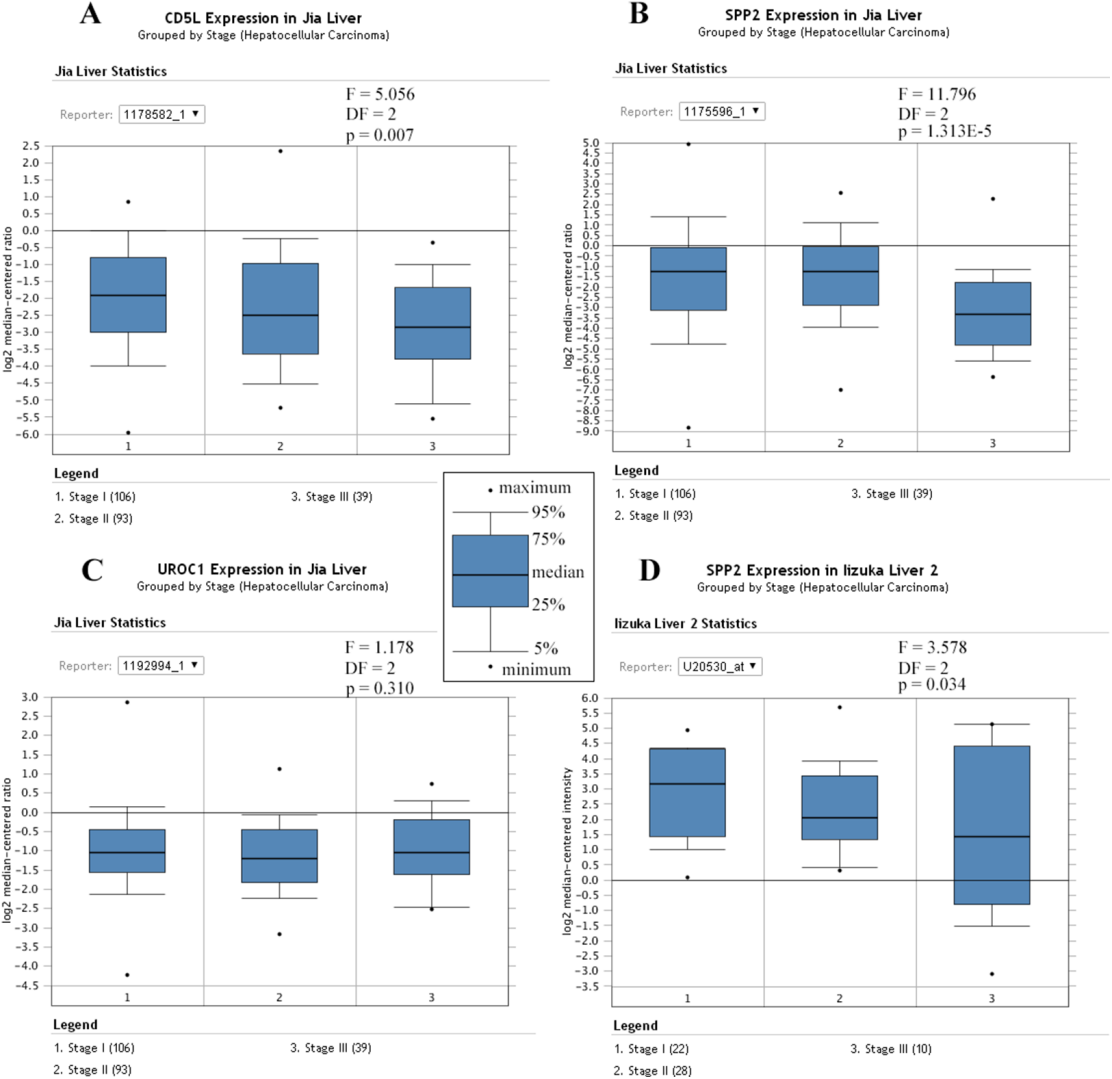
Differential expression profiles of the SKGs in HCCs of different stages. (A) Differential expressions of CD5L in HCCs of different stages; (B) and (D) Differential expressions of SPP2 in HCCs of different stages; (C) No significant difference of UROC1 expression in HCCs of different stages. SKGs, special key genes. One-way ANOVA analysis was used and *p* < 0.05 was considered to be statistically significant.

Similarly, Jia liver (Jia et al., 2007) and Iizuka Liver 2 (Iizuka et al., 2003) were selected for the differential analysis of the SKGs among HCCs of different stages. Although four genes (CD5L, IGF2, UROC1, and SLC22A10) were shown to be correlated with HCC-A stage in above analyses, only CD5L, IGF2, and UROC1 were analyzed for further validation since no data were found for SLC22A10 in the two datasets. As CD5L and UROC1 were only shown in Jia liver, they were analyzed in this dataset only. Through ANOVA analysis, CD5L and SPP2 were shown to be differentially expressed among HCCs of different stages while no significant difference of UROC1 was shown (Fig. 10).

**Figure 11:**
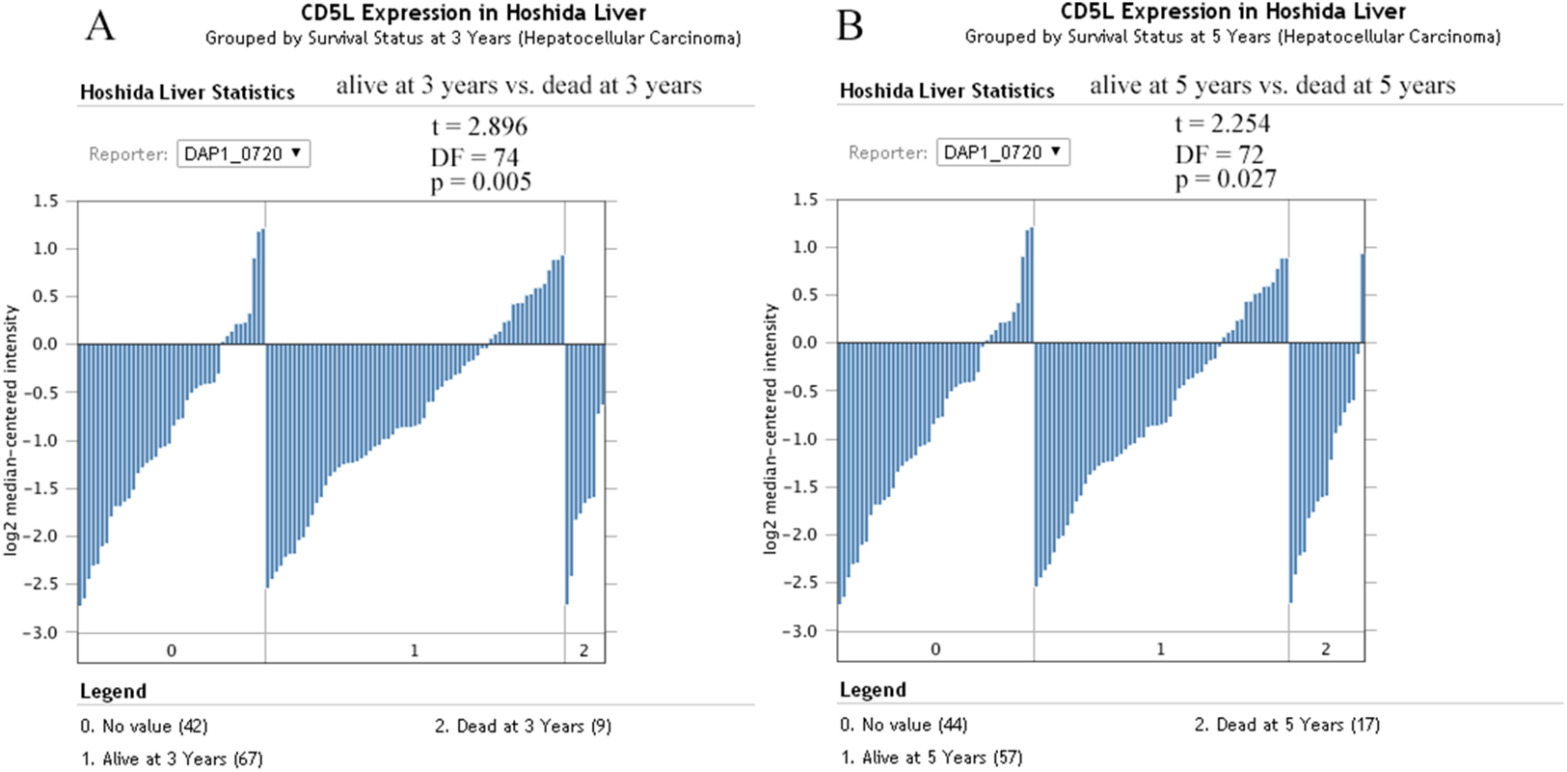
The effects of CD5L expression on overall survival of HCC patients. (A) CD5L is higher expressed in the alive patients than the dead ones at 3 years; (B) CD5L is higher expressed in the alive patients than the dead ones at 5 years.

For survival analysis, only the dataset Hoshida Liver (Hoshida et al., 2009) was eligible and only the overall information was provided. As shown in Fig. 11, CD5L was higher in the patients which were alive at 3 and 5 years than the dead ones which was consistent with its favorable prognostic role for overall survival of HCC patients in the above analysis.

## Discussion

Considering the poor prognosis of HCC due to its later diagnosis, identification of stage-associated markers for HCC is very important. As different etiologies might have different effects, the HCCs with different risk factors might have different gene expression profiles which would further lead to different development processes of HCC. Considering the important role of alcohol in HCC development, identification of the key genes during alcohol-related HCC development was very crucial. In this study, we confirmed the impact of risk factors (grouping) on the overall survival of HCC patients. Multivariable stepwise Cox regression had been shown to be effective to find independent prognostic factors in many studies (Arima et al., 2015; Esteban et al., 2015; Shah et al., 2014; Zemmour et al., 2015). Here, through this method, pathological stage was shown to be independent unfavorable prognostic factor for overall and disease-free survival of HCC patients, indicating the importance of earlier diagnosis for good prognosis. HCC-A patients were found to be diagnosed later than the HCC patients with other risk factors and 10 genes were identified to be SKGs for alcohol-related HCCs which were differentially expressed between HCC-A and at least one of other HCCs groups. The differential expression profiles of the SKGs in HCC were confirmed and their clinical significances were also validated through other HCC datasets.

CD5L, one of the down-regulated SKGs, was identified in 1997 (Gebe et al., 1997). Given its anti-apoptotic role on leukocytes, it was considered to be an emerging key component among the repertoire of immune effectors (Sanjurjo et al., 2015). Although CD5L was reported to be increased in HCV-related fibrosis and HCV-induced cirrhosis, the few samples (four patients each group) reduced the reliability of the results (Gangadharan et al., 2007). In this study, down-regulation of CD5L in alcohol-related HCC patients was shown, indicating its potential anti-tumor role in alcohol-related HCC development. Through multivariable Cox regression analysis, CD5L was shown to be independent favorable prognostic factor for overall survival of HCC-A patients. But it was noticeable that the prognostic role of CD5L disappeared when pathological stage was used as strata variable, it was indicated that the effects of CD5L on overall survival was associated with its correlation with the tumor stage. The decreased expression of CD5L in later stages and its favorable prognostic role for HCC patients were also shown in HCCs without grouping and other HCC datasets in this study, indicating its crucial role in HCC development and progression. As different factors might have different effects on HCC process, its roles in alcohol-related HCCs and HCCs with other risk factors needed to be investigated further.

Among the down-regulated SKGs, other three down-regulated genes (SLC22A10, UROC1, and SPP2) were shown to be negative correlated with both pathological stage and pathological grade of HCC-A tumors. As liver enriched genes, their down-regulation in HCCs might have some important effects. SLC22A10, also known as OAT5, was a member of the organic anion transporters (OATs) which could transport small hydrophilic anions and diuretics, non-steroidal anti-inflammatory drugs, anti-tumor drugs antibiotics, and antiviral nucleoside analogs across membrane barriers of epithelia of diverse organs (Klein et al., 2010). Studies on SLC22A10 were mainly on kidney disease, especially on the nephrotoxicity of the drugs (Bulacio et al., 2015; Bulacio & Torres, 2015). To our knowledge, this was the first study about SLC22A10 on HCC. Considering its essential role in the elimination of numerous endogenous and exogenous organic anions from the body, the decrease of SLC22A10 might reduce its clearance function. Its negative correlation with the HCC-A stage and grade might provide some new clues for the mechanism of alcohol-related HCC development. As its down-regulation was also obvious in the later stages of HCCs (overall), its role in HCC progression needed more investigation.

The gene UROC1 coded the enzyme urocanase which could catalyzes the second step in the degradation of histidine, the hydration of urocanate into imidazolonepropionate (Kessler, Rétey & Schulz, 2004). Here, we found its down-regulation in alcohol-related HCCs and its negative correlation with the HCC-A stage, indicating its important role during alcohol-related HCC development. Its down-regulation in HCC patients (overall) of later stages also indicated its potential role in HCC progression. Considering there were few studies about UROC1 in cancer, its special functions in HCC development needed further investigation.

SPP2 coded the secreted phosphoprotein 24 KD (Spp24), a member of the cystatin superfamily, which was shown to be important in bone metabolism (Murray et al., 2015). Recently, its anti-tumor activity was reported in prostate cancer cells, pancreatic cancer cells and lung cancer cells (Lao et al., 2016; Li et al., 2015; Murray et al., 2015). In liver cancer, it was reported to be down-regulated in HBV-related HCCs (Yang et al., 2018). Here, although the expressional difference of SPP2 was not confirmed in the paired HCC-A patients, it was lower in most of the HCC-A tumors than their paired normal controls and its correlation with HCC-A stage was obvious. Through the Oncomine database, one HCC dataset showed its up-regulation while other four datasets showed its down-regulation, indicating some heterogeneity in its expressions. However, considering its negative correlation with stage and grade of HCC-A tumors and its obvious down-regulation in HCCs of later stages, it might be new target for treatment of alcohol-related HCCs and progressed HCCs.

CSAG3, also known as TRAG-3 (taxol resistance associated gene-3), was reported to be up-regulated in many tumors including gastric cancer, urothelial carcinoma of the bladder, ovarian carcinoma, and melanoma (Aung et al., 2006; Feller et al., 2000; Karam et al., 2011; Materna et al., 2007). As one of the SKGs in this study, its up-regulation in alcohol-related HCCs and positive correlation with HCC-A grade was shown. To our knowledge, the dysfunction of CSAG3 was first reported in alcohol-related HCCs in this study and this result might provide new clues for the study of alcohol-related HCC development.

CSMD1, another up-regulated gene of the SKGs, was also shown to be correlated with HCC-A grade in this study. It was noticeable that CSMD1 was reported to be an important oncosuppressor in many tumors including melanoma (Tang et al., 2012), breast cancer (Kamal et al., 2010), head and neck cancer (Scholnick & Richter, 2003), and colorectal adenocarcinomas (Farrell et al., 2008). When it came to HCC, the role of CSMD1 was still controversial. In one study, down-regulated expression of CSMD1 by miR10b in HCC cell lines was reported to be associated with HCC cell viability and invasion (Zhu et al., 2016). But in another study, over-expression of CSMD1 was found in dysplastic liver nodules and HCCs (Frau et al., 2012). Here, up-regulated CSMD1 was found in alcohol-related HCC patients and two other HCC datasets also showed its up-regulation in HCC patients. For its clinical significance, CSMD1 was shown to be positively correlated with HCC-A grade and its independent unfavorable prognostic factor for HCC-A disease-free survival was also indicated. Although CSMD1 was not shown significantly differentially expressed in TCGA HCCs (overall) and the normal liver controls, the prognostic role for disease-free survival was shown. Since the specific function of CSMD1 in HCC is not yet clear enough, our results might provide some new clues for the study of HCCs, especially alcohol-related HCCs.

During our analysis, we considered the potential effects of different risk factors and grouped the patients according to their pathological stage and risk factors. We also investigate the expressional differences of the SKGs in HCCs (overall). Considering that different risk factors might have different effects on HCCs, the difference in gene expression profiles under specific risk factors might be obscured when not grouping. So, it was not surprising to find that the differences of six of the genes were not so significant in HCCs (overall) as that in alcohol-related HCCs. Similarly, considering the stage difference between the HCC-A group and HCC-V group, it was also not surprising to see the four genes which were shown to be negatively correlated with the HCC-A stage were all shown to be differentially expressed in HCCs (overall) of different stages. Through the analysis above, it was indicated that grouping patients according to the risk factors to find marker for related patients and grouping patients according to the stages would be useful to find markers associated with tumor progression.

## Conclusions

In summary, we identified 10 genes as SKGs for alcohol-related HCC in this study. Among the SKGs, four genes (CD5L, SLC22A10, UROC1, and SPP2) were shown to be negative correlated with HCC-A pathological stage and their correlations with HCC stage were confirmed in other HCC datasets, indicating that they might be new makers for HCC progression, especially alcohol-related HCC progression. Five genes of the SKGs were shown to be correlated with HCC-A grade. They might provide new clues for the study of alcohol-related HCC development. In addition, we identified CD5L as the favorable prognostic factor for overall survival and CSMD1 as the unfavorable prognostic factor for disease-free survival for HCC-A patients and HCC patients in whole. They might be new prognostic markers for HCCs, especially alcohol-related HCCs. However, considering some of the SKGs were reported in our study for the first time and the analyses were based on gene expression levels, large scale investigation was also needed to confirm their specific roles in alcohol-related HCC development and HCC progression.

## Additional Information and Declarations

### Data Availability

The following information was supplied regarding data availability: raw data were generated in the study. All the original data in this study weredownloaded from the public databases including TCGA (https://portal.gdc.cancer.gov/), GEO (https://www.ncbi.nlm.nih.gov/geo/) and Oncomine (https://www.oncomine.org/). For the SKGs identification, we used TCGA-LIHC (https://portal.gdc.cancer.gov/projects/TCGA-LIHC) and GSE59259 (https://www.ncbi.nlm.nih.gov/geo/query/acc.cgi?acc=gse59259). The HCC datasets from the Oncomine database (https://www.oncomine.org) were: Chen liver, Mas liver, Roessler liver, Roessler liver 2, Wurmbach liver, Jia liver, and Iizuka Liver 2.
